# *Pimelodus maculatus* (Siluriformes, Pimelodidae): complete mtDNA sequence of an economically important fish from the São Francisco river basin

**DOI:** 10.1080/23802359.2016.1219646

**Published:** 2016-11-22

**Authors:** Leonardo Cardoso Resende, Anderson Oliveira do Carmo, Daniela Núñez-Rodriguez, Juliana da Silva Martins Pimentel, Alessandra Gomes Bedore, Hortênsia Gomes Leal, Evanguedes Kalapothakis

**Affiliations:** a Biologia Geral, Universidade Federal de Minas Gerais Instituto de Ciencias Biologicas, Belo Horizonte, Brazil;; b Departamento de Biologia Geral, Instituto de Ciências Biológicas, Universidade Federal de Minas Gerais, Belo Horizonte, Brazil

**Keywords:** Complete mtDNA, ‘mandi’, ‘yellow-mandi catfish’, next-generation sequencing, *Pimelodus maculatus*

## Abstract

Pimelodus *maculatus* is an important commercial fish found in the São Francisco and Paraná river basins. NGS was used to sequence the mtDNA of *P. maculatus*. The mtDNA was annotated and aligned with that of 25 other fish species to enable phylogenetic analysis. The complete mtDNA molecule had 16,561 bp and its GC content was 43.7%; the structure was similar to that of other vertebrates: 2 rRNA, 22 tRNA, 13 protein-coding genes, and a D-loop region containing 914 bp. Phylogenetic analysis yielded a tree with a high bootstrap coefficient that was coherent with the actual phylogeny of the species.

Pimelodidae is the richest family in the order Siluriformes and it is composed of 90 valid species described throughout South America. The majority of pimelodid species are endemic to the neotropics; *Pimelodus maculatus* is distributed in Brazil throughout the Paraná River Basin (PRB) and the São Francisco River Basin (SFRB). Commonly known as ‘yellow-mandi catfish’ or ‘mandi amarelo’, *P. maculatus* is a migratory fish with socioeconomic relevance based on its size (maximum standard length, 36 cm) (Lundberg & Littmann 2003) and its frequency in artisanal fisheries in PRB and SFRB (Franco de Camargo & Petrere 2001; Agostinho & Gomes 2002). Thus, it is not listed as threatened or vulnerable species on the International Union for Conservation of Nature and Natural Resources Red List (IUCN 2016) or in Brazilian laws (MMA 2014).

Several genetic studies of partial mitochondrial and nuclear regions of *P. maculatus* have found a high degree of genetic diversity among populations (Garcia & Moreira Filho 2005; Paiva & Kalapothakis 2008; Carvalho et al. 2011; Lundberg et al. 2011; Pereira et al. 2011; Ribolli et al. 2012; Ferreira et al. 2014; Frantine-Silva et al. 2015). Here, we provide the first description of the complete mitochondrial DNA (mtDNA) sequence for *P. maculatus*, which could support the development of molecular markers studies to aid wildlife forensics, evolutionary approaches, and conservation and management studies.

One *P. maculatus* specimen was collected in the SFRB, Minas Gerais, Brazil (19°57′07″S, 44°20′34″W). Muscle tissue was extracted and stored at the Tissue and DNA Collection facility of the Universidade Federal de Minas Gerais (deposit code: UFMG-BDT-PP000006). A genomic library was constructed using the Nextera DNA Library Preparation kit (Illumina Inc., San Diego, CA) and sequenced using a MiSeq sequencer (Illumina), with a paired-end 300 bp strategy. The CLC Workbench software (ver. 9.0; CLC Bio-Qiagen, Aarhus, Denmark) was used for *de novo* assembly, and the mitochondrial genome was annotated and analyzed using the MitoFish webserver (Iwasaki et al. 2013). Complete mtDNA sequences from 25 other species, available from GenBank, were aligned to enable phylogenetic analysis with the MEGA software (version 7.0.14; Kumar et al. 2016), using a maximum likelihood method with 1000 bootstrap replications and the Tamura–Nei model (Tamura & Nei 1993) for nucleotide substitution (Figure 1).

**Figure 1.**
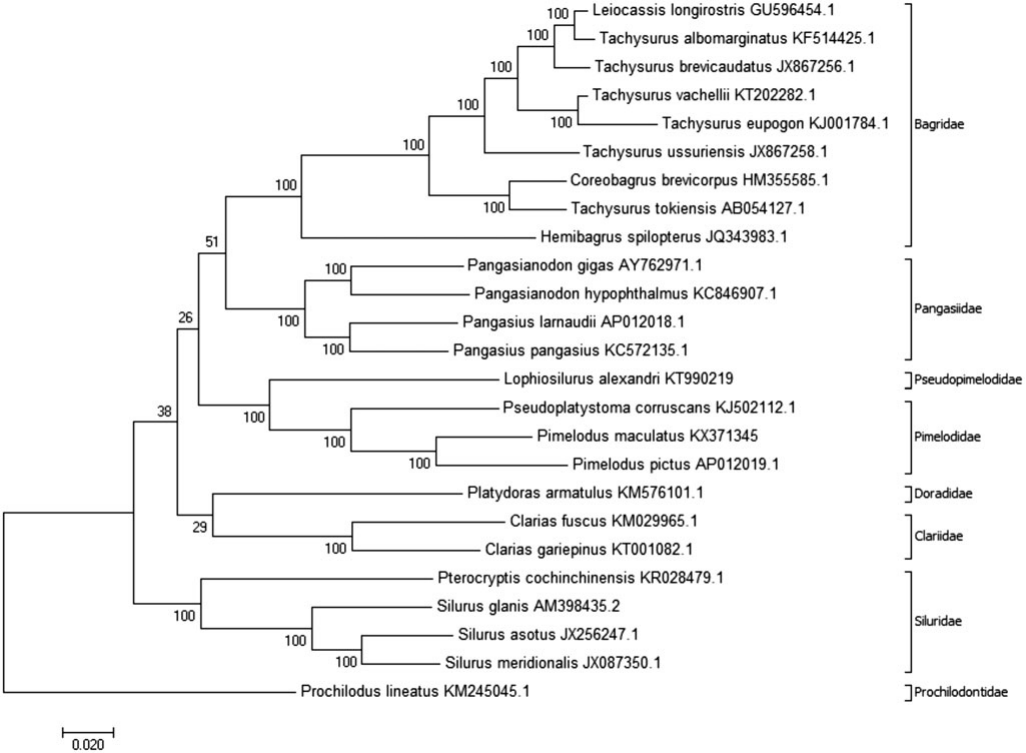
Molecular phylogenetic analysis inferred using the maximum likelihood method based on the Tamura–Nei model (Tamura & Nei 1993) with 1000 bootstrap replications. The analysis was carried out using complete mtDNA of *Pimelodus maculatus* (GenBank accession no. KX371345) and complete mtDNA of 25 other species: *Leiocassis longirostris* (GU596454.1), *Tachysurus albomarginatus* (KF514425.1), *Tachysurus brevicaudatus* (JX867256.1), *Tachysurus vachellii* (KT202282.1), *Tachysurus eupogon* (KJ001784.1), *Tachysurus ussuriensis* (JX867258.1), *Coreobagrus brevicorpus* (HM355585.1), *Tachysurus tokiensis* (AB054127.1), *Hemibagrus spilopterus* (JQ343983.1), *Pangasianodon gigas* (AY762971.1), *Pangasianodon hypophthalmus* (KC846907.1), *Pangasius larnaudii* (AP012018.1), *Pangasius pangasius* (KC572135.1), *Lophiosilurus alexandri* (KT990219), *Pseudoplatystoma corruscans* (KJ502112.1), *Pimelodus pictus* (AP012019.1), *Platydoras armatulus* (KM576101.1), *Clarias fuscus* (KM029965.1), *Clarias gariepinus* (KT001082.1), *Pterocryptis cochinchinensis* (KR028479.1), *Silurus glanis* (AM398435.2), *Silurus asotus* (JX256247.1), *Silurus meridionalis* (JX087350.1), and *Prochilodus lineatus* (KM245045.1). The phylogenetic tree with the highest log likelihood is shown, with the percentages of trees in which associated taxa clustered together shown next to the branches. The phylogenetic tree was rooted with *P. lineatus* (Characiformes, Prochilodontidae). The D-loop region was excluded from this analysis because it is considered to be highly variable (Gonder et al. 2007). The phylogenetic tree obtained was coherent with the phylogenetic studies of Sullivan et al. (2006, 2013) and Lundberg et al. (2011), which grouped species in their respective families; Pimelodidae and Pseudopimelodidae were grouped together in the superfamily Pimelodoidea. The analyses were conducted using MEGA7 software (Kumar et al. 2016).

The mtDNA sequence (16,561 bp, 514.9 folds of coverage) was deposited in GenBank (accession no. KX371345). The GC content of the sequence was 43.7% and the base frequencies were 31.2% A, 15.5% G, 25.0% T, and 28.2% C. The structure has 2 rRNAs, 22 tRNAs, 13 protein-coding genes, and a 914-bp D-loop. The *ND6* gene and regions tRNA-Ala, t-RNA-Asn, tRNA-Cys, tRNA-Tyr, tRNA-Ser, tRNA-Glu, and tRNA-Pro were coded on the light strand. The other genes, tRNA and rRNA, were coded on the heavy strand. Seven regions (*ND2*, *COII*, *Atp6*, *COIII*, *ND3*, *ND4*, and *Cyt b*) had incomplete stop codons, which were completed in post-transcriptional polyadenylation (Ojala et al. 1981), and four tRNAs had different anticodons (UAA and UAG to leucine, and UGA and GCU to serine).
